# Gestational age acceleration is associated with epigenetic biomarkers of prenatal physiologic stress exposure

**DOI:** 10.1186/s13148-022-01374-9

**Published:** 2022-11-28

**Authors:** Verônica Euclydes, Catarina Gomes, Gisele Gouveia, Vinicius Daguano Gastaldi, Arthur Sant’Anna Feltrin, Caroline Camilo, Rossana Pulcineli Vieira, Aloísio Felipe-Silva, Sandra Grisi, Günther Fink, Alexandra Brentani, Helena Brentani

**Affiliations:** 1 Department and Institute of Psychiatry, University of São Paulo Medical School, Rua Dr. Ovídio Pires de Campos, 785, LIM23 (Térreo), São Paulo, 05403-010 Brazil; 2 Laboratório de Psicopatologia e Terapêutica Psiquiátrica (LIM23), Faculdade de Medicina FMUSP, Universidade de Sao Paulo, Sao Paulo, Brazil; 3 Center of Mathematics, Computation and Cognition, Federal University of ABC, São Bernardo do Campo, Brazil; 4 Departamento de Obstetrícia e Ginecologia, Faculdade de Medicina FMUSP, Universidade de Sao Paulo, Sao Paulo, SP Brazil; 5 Departamento de Patologia, Hospital Universitário, Universidade de Sao Paulo, Sao Paulo, SP Brazil; 6 Department of Epidemiology and Public Health, Swiss Tropical and Public Health Institute, University of Basel, Basel, Switzerland; 7 Departamento de Pediatria, Faculdade de Medicina FMUSP, Universidade de Sao Paulo, Sao Paulo, SP Brazil

**Keywords:** DNA methylation age, Gestational age acceleration, Prenatal psychosocial stress, Sex bias

## Abstract

**Background:**

Physiological maternal stress response, such as imbalance in the glucocorticoid pathway and immune system seems to be mediated by DNA methylation (DNAm) and might translate intrauterine stress exposures into phenotypic changes in a sex-specific manner. DNAm in specific sites can also predict newborn gestational age and gestational age acceleration (GAA). GAA occurs when the predicted biological age is higher than the chronological age. In adults, poor health outcomes related to this deviance are well documented and raise questions for the interpretation and prediction in early stages of life. Boys seem to be more vulnerable to intrauterine stress exposure than girls; however, the mechanisms of adaptive sex-specific responses are still unclear. We hypothesize that intrauterine stress exposure is associated with GAA and could be different in boys and girls if inflammatory or glucocorticoid pathways exposure is considered.

**Results:**

Using the Western Region Birth Cohort (ROC—São Paulo, Brazil) (n = 83), we calculated DNAm age and GAA from cord blood samples. Two epigenetic risk scores were calculated as an indirect proxy for low-grade inflammation (i-ePGS) and for glucocorticoid exposure (GES). Multivariate linear regression models were applied to investigate associations of GAA with prenatal exposures. The i-ePGS and GES were included in different models with the same co-variates considering sex interactions. The first multivariate model investigating inflammatory exposure (adj. R^2^ = 0.31, *p* =  < 0.001) showed that GAA was positively associated with i-ePGS (CI, 0.26–113.87, *p* = 0.049) and negative pregnancy-related feelings (CI, 0.04–0.48 *p* = 0.019). No sex interaction was observed. The second model investigating glucocorticoid exposure (adj. R^2^ = 0.32, *p* =  < 0.001) showed that the higher was the GAA was associated with a lower the lower was the GES in girls (CI, 0.04–2.55, p = 0.044). In both models, maternal self-reported mental disorder was negatively associated with GAA.

**Conclusion:**

Prenatal epigenetic score of exposure to low-grade inflammatory was a predictor of GAA for both sexes. Glucocorticoid epigenetic score seems to be more important to GAA in girls. This study supports the evidence of sex-specificity in stress response, suggesting the glucocorticoid as a possible pathway adopted by girls to accelerate the maturation in an adverse condition.

**Supplementary Information:**

The online version contains supplementary material available at 10.1186/s13148-022-01374-9.

## Background

The identification of intrauterine stress biomarkers is fundamental in the context of early interventions targeting poor health outcomes prevention later in life. A growing body of research suggests the biological pathways, such as the glucocorticoid and pro-inflammatory pathways that could be the translators of an environmental experience into phenotypic changes mediated by the epigenome [1–3]. Molecular mechanisms related to cells plasticity, such as DNA methylation (DNAm) levels, may mediate the activation of pathways related to physiological stress due to its dynamic interaction with the environment [4, 5]. To detect overexposure to inflammation or glucocorticoids during gestation requires complex study designs and it is challenging matter. As DNAm presents marks of environmental experiences, some studies have proposed epigenetic scores that can reflect these exposures [6, 7]. DNAm in specific CpGs can also be used to build epigenetic clocks capable of predicting the chronological age of a sample with relative accuracy [5, 8]. Importantly, a higher estimation of age by DNAm compared to chronological age (age acceleration) is associated with age-related diseases in adult life [5, 9, 10]. Age acceleration has been suggested as a candidate biomarker of intrauterine stress, measured as a gestational age acceleration (GAA) [11, 12]. Additionally, adaptive stress responses are sex-specific, as well as the methylation patterns across the genome [13]. Previous results provide evidence of differences regarding the HPA axis and inflammatory pathways in males and females, but it is not clear if integrating environmental stressors and biomarkers of endogenous stress could predict GAA differentially in boys and girls.

Glucocorticoid and inflammatory pathways are known to play a key role in fetal development [3, 14, 15]. Specifically, glucocorticoid participates in decidualization, implantation, placentation, fetal brain development, lung maturation, and parturition [15, 16]. However, overexposure to glucocorticoids can lead to fetal and placental growth restriction, compromising fetal development [17]. Prenatal maternal distress may lead to alterations in neonatal hippocampal connectivity through the hypothalamic-pituitary adrenal (HPA) axis and glucocorticoid signaling [18, 19]. Inflammatory pathways are also critical and fundamental for fetal development, but overexposure to inflammatory markers appears to be predictors of worse obstetric and postnatal outcomes [20, 21]. Epigenetic mechanisms have been proposed as mediators in this context for a long time [3, 6]. Barker et al. [7] replicate an inflammation epigenetic polygenic score (i-ePGS), as a marker of low-grade inflammation exposure in a developmentally sensitive framework. They showed an association of i-ePGS with externalizing and cognitive symptoms later in life, supporting a link between gestational inflammation exposure and child and adolescent mental health outcomes.

Clinical measurements as birth weight and gestational age are known factors that reflect intrauterine quality of life and are also predictors of health outcomes, including chronic diseases. DNAm patterns have been associated with different types of stress exposure and gestational age [22].Recently, Bohlin et al.[23] and Knight et al.[24] developed algorithms from DNAm patterns in cord blood to obtain DNAm age, including estimates of epigenetic gestational age. Knight et al. reported that GAA significantly predicted birth weight, which was confirmed in the Avon Longitudinal Study of Parents and Children (ALSPAC) and extended to birth length [25]. Two studies showed that neonates in the highest birth weight percentiles presented GAA while neonates falling in the lowest percentiles presented deceleration (i.e., when chronological age is higher than epigenetic age [12, 26]. Furthermore, GAA was observed in newborns from low socioeconomic status women, with Sjögren syndrome, insulin-treated gestational diabetes mellitus, and experiencing antenatal depressive symptoms [12].

Such findings suggest that GAA or deceleration could represent different possible answers in newborns exposed to prenatal stressors. This has been confirmed by a study that measures cerebroplacental ratio (CPR), a hemodynamic parameter reflecting fetal adaptation to hypoxic conditions, which was associated with epigenetic age acceleration [27]. Specifically, subjects exhibiting decreased CPR, and exposed to prenatal adverse conditions, were born with decelerated epigenetic age. Knight et al. [28] showed that GAA was inversely correlated with the necessary number of respiratory interventions and outcome’s severity in extremely preterm infants, suggesting that GAA could be a metric of developmental maturity. The authors found a higher GAA in girls which could suggest a higher maturation. They suggested that the lack of associations with neonatal interventions in the analyses stratified by sex could be due to a reduction in power from a decreased sample size.

A broad range of studies, based on the sex bias of prevalence of diseases and disorders, points to a sex-specific differences in stress response. We and others [29, 30] have provided evidence of sex bias in cord blood DNA methylation. Recently McGill et al. [6] showed that prenatal maternal anxiety predicted infant AA (PedBE algorithm) in two cohorts, independent of obstetric, socioeconomic, and genetic risk factors. Also, AA predicted increased externalizing symptoms in males from mid- to late childhood in one of the two cohorts. Previously Binder et al. [31] suggested that age acceleration is associated with faster puberal development in girls. In this context, we hypothesize that GAA could represent a tentative adaptation/maturation of the fetus in face of intrauterine stress exposure, which is differently influenced in males and females depending on glucocorticoid or inflammation pathways exposure. To explore our goal, we performed multivariate regression models using prenatal stress exposure variables, and two epigenetic scores associated with cortisol and inflammation levels of exposure as predictors of GAA with sex as a moderator.

## Results

### Sample characterization

Table 1 shows the maternal and the newborn characteristics. From a total of 96 cord blood collected we excluded 14 due to incomplete questionnaire data or being twinned. The average maternal age of participants was 25 years, and the majority presented pre-gestational overweight (BMI > 25 kg/m^2^), according to World Health Organization (WHO) classification. Sixteen women (9.6%) reported smoking cigarettes during pregnancy, 13% reported mental disorders. For the newborns, 54.2% were female, 6% were preterm (GA < 37 weeks) and the average weight and length at birth were 3324 g and 49 cm, respectively. The average GA estimated by the Capurro method was 39 weeks.

**Table 1:** Summary descriptive table of the sample compared by sex

	Boys (*N* = *37*)	Girls (*N* = *45)*	*p* value
***Maternal exposures***			
Mother age	24.9 (6.41	26.6 (7.19)	0.254
Perceived stress in relation to pregnancy	1.78 (1.16)	1.89 (1.01)	0.666
*Educational level (mother):*			
1–2	21 (60.0%)	24 (55.9%)	0.454
3–4	11 (31.4%)	12 (27.9%)	
5	3 (8.57%)	1 (2.33%)	
Relationship with the father (months)	59.7 (51.3)	66.1 (50.1)	0.575
*Smoking:*			
No	5 (13.5%)	11 (24.4%)	0.336
Yes	32 (86.5%)	34 (75.6%)	
*Mental disorder:*			
No	37 (100%)	39 (86.7%)	**0.030**
Yes	0 (0.00%)	6 (13.3%)	
***Newborn outcomes***			
Gestational Age (weeks)	39.4 (1.81)	39.2 (1.35)	0.693
*Preterm*			
No	34 (91.9%)	43 (95.6%)	0.654
Yes	3 (8.11%)	2 (4.44%)	
Length at birth	49.4 (1.80)	47.9 (2.14)	0.001
Birth weight	3397 (510)	3251 (485)	0.191
Thoracic circumference	33.4 (1.83)	33.1 (1.65)	0.459
Abdominal circumference	32.5 (2.17)	32.2 (2.20)	0.618
Cephalic circumference/Age (HCZ)	0.27 (1.03)	0.20 (0.94)	0.723
Height/Age (HAZ)	−0.28 (0.95)	−0.68 (1.15)	0.093
Weight/Age (WAZ)	0.05 (1.03)	−0.01 (1.03)	0.796
BMI/Age (BAZ)	0.28 (1.22)	0.55 (1.09)	0.301
DNAm GA (Knight et al.)	38.9 (1.32)	38.9 (1.04)	0.845
DNAm GA (Bohlin et al.)	39.4 (0.65)	39.5 (0.70)	0.839
GAA by Bohlin et al	−0.05 (0.86)	0.04 (1.12)	0.679
GAA by Knight et al	−0.54 (1.79)	−0.34 (1.61)	0.611
*Direction of GAA*			
Negative	18 (48.6%)	20 (44.4%)	0.875
Positive	19 (51.4%)	25 (55.6%)	
i-ePGS	−0.035	−0.034	0.337
Glucocorticoid CpGs (GES)	−0.96 (0.40)	−0.94 (0.32)	0.856

Bold means* p*-value < 0.05

### Gestational age acceleration and birth outcomes

The epigenetic clock developed by Bohlin was chosen over the one developed by Knight because of the higher correlation with clinical GA (0.43 vs. 0.26, respectively). We did not observe mean differences or statistically significant correlation between maternal exposures or neonatal anthropometry among the individuals who presented GAA (Additional files 1 and 2: Fig. S1 and Table S1). Furthermore, in the bivariate analysis, newborn anthropometry presented no associations with GAA (Additional file 3: Table S2).

### Age acceleration is related to maternal stress exposures, GES and i-ePGS

**Fig. 1:**
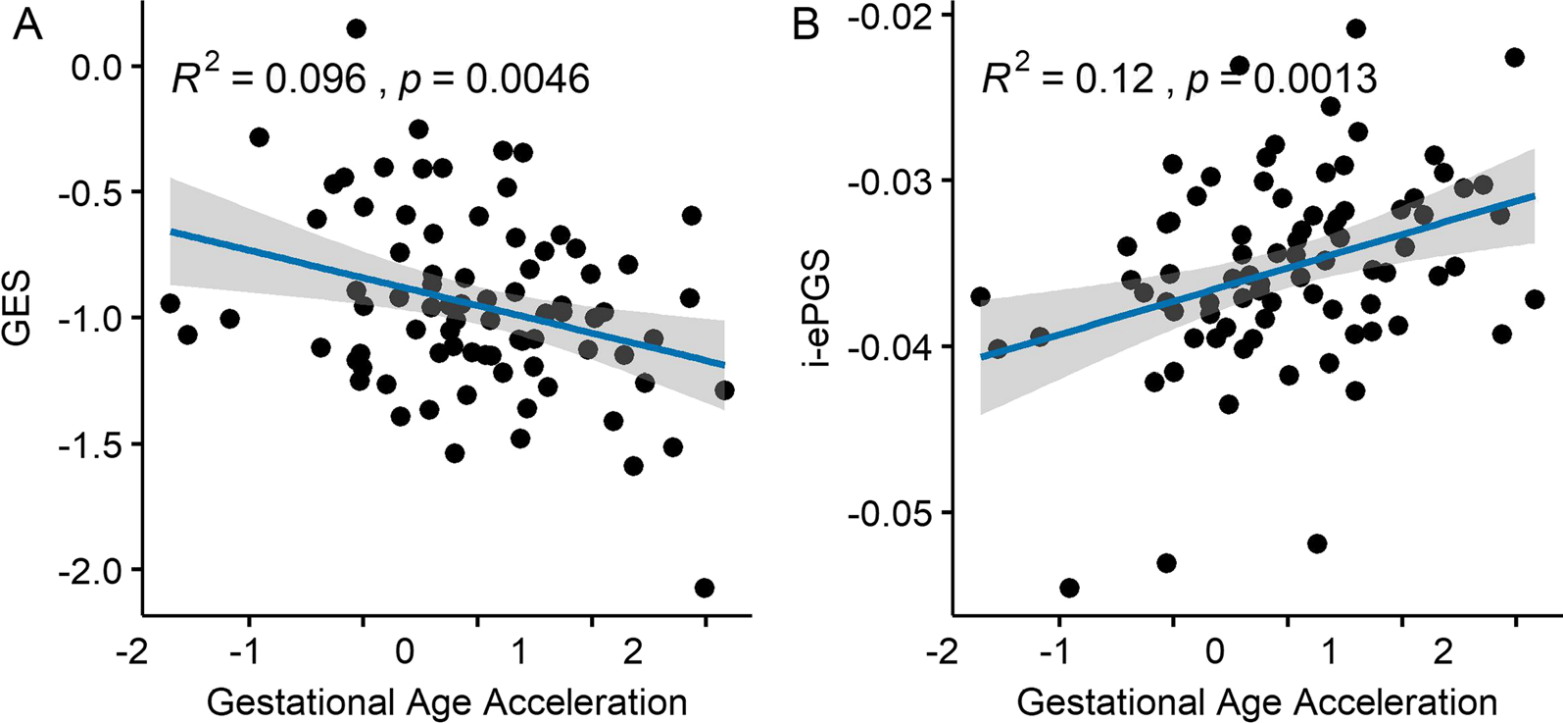
**A** Association between GAA and GES (*β* =  − 0.88; *p* = 0.005). **B** Associations between GAA and i-ePGS (*β* = 60.4; *p* = 0.001)

Our main objective was to understand whether environmental maternal–fetal exposures were predictors of the GAA and if sex could interact with this outcome. We analyzed psychosocial and candidate biomarkers for low grade inflammation and glucocorticoid levels of exposure. We interpret birth anthropometric variables as a proxy of the intrauterine environment quality as it has been used as a proxy of fetal growth in DOHaD studies, reflecting the intrauterine milieu condition. GAA was negatively associated with mean DNAm levels on Glucocorticoid Epigenetic Score (GES) CpGs (β = −0.88; *p* = 0.005) (Fig. 1A) and positively associated with i-ePGS (β = 60.4; *p* = 0.002) (Fig. 1B). GAA was not correlated with psychosocial variables individually (Additional file 1: Figs. S1 and S2).

Maternal age, familial income, educational levels, smoking, mental disorder, self-report feelings regarding pregnancy, time in the relationship with the father (months), and infant anthropometry were included in a multivariate analysis. The i-ePGS and GES were included in separate regression models (Tables 2 and 3, respectively**)**. Importantly, we investigated sex interactions between the epigenetic predictors and the outcome.

**Table 2:** Multivariate model with GAA as outcome and i-ePGS as low-grade inflammation epigenetic exposure score

Predictors	Gestational Age Acceleration (GAA)		
	Estimates	CI	*p* value
(Intercept)	3.08	0.66–5.51	**0.014**
i ePGS	57.07	0.26–113.87	**0.049**
i ePGS * sex [GIRLS]	−38.97	−107.24 to 29.29	0.257
Sex (Girls)	−1.30	−3.75 to 1.15	0.293
Mother age	−0.03	−0.07 to 0.00	**0.036**
Perceived stress in relation to pregnancy	0.26	0.04–0.48	**0.019**
Mental disorder	−0.77	−1.50 to −0.03	**0.042**
Time in relationship with father	−0.00	−0.01to 0.00	0.730
Familial income	0.11	−0.12 to 0.33	0.348
Mother education	−0.10	−0.33 to 0.12	0.354
Smoking during Pregnancy	0.01	−0.56 to 0.58	0.974
Cephalic circumference (z-score)	0.24	−0.02 to 0.50	0.074
Weight for Age (z-score)	0.02	−0.26 to 0.29	0.893
Cell Composition	0.10	−0.02 to 0.22	0.109

Bold values means* p* < 0.05

Observations: 68

Asterisk means moderation model

R^2/^R^2^ adjusted: 0.443/0.308

**Table 3:** Multivariate model with GAA as outcome and GES as glucocorticoid exposure epigenetic exposure score

Predictors	Gestational age acceleration (GAA)		
	Estimates	CI	*p* value
(Intercept)	1.24	−4.02 to 6.49	0.640
GES	0.38	−4.56 to 5.32	0.878
GES* sex [GIRLS]	1.29	0.04 to 2.55	**0.044**
Sex (Girls)	1.36	0.12 to 2.60	**0.032**
Mother Age	−0.03	−0.06 to 0.00	0.053
Perceived stress in relation to pregnancy	0.18	−0.04 to 0.40	0.098
Mental disorder	−0.85	−1.60 to −0.11	**0.026**
Time in relationship with father	−0.00	−0.01 to 0.00	0.468
Familial income	0.10	−0.12 to 0.33	0.361
Mother education	−0.07	−0.30 to 0.15	0.513
Smoking during pregnancy	0.25	−0.33 to 0.83	0.389
Cephalic circumference (z-score)	0.30	−0.03 to 0.58	**0.032**
Weight for age (z-score)	0.07	−0.18 to 0.32	0.569
Cell composition	0.32	−0.49 to 1.13	0.433

Bold values means* p* < 0.05

Observations: 68

Asterisk means moderation model

R^2/^R^2^ adjusted: 0.449/0.316

The multivariable regression model, including the i-ePGS as a predictor, shows a positive association with GAA residuals (CI, 0.26–113.9, *p* = 0.049). Younger mothers presented higher GAA. Regarding the psychosocial variables, GAA showed a positive association with negative feelings related to pregnancy (CI, 0.04–0.48, *p* = 0.019) and psychiatric disorder (CI, −1.50 to −0.03, *p* = 0.042). Sex interactions were not observed in the i-ePGS.

In the final multivariable regression model including the GES as predictor, GAA residuals showed a positive association with GES in a sex-specific pattern (CI, 0.04:2.55, *p* = 0.044 for girls). The GAA was also associated with mental disorder (CI, −1.60: −0.11, *p* = 0.005).

The glucocorticoid pathway is known to be associated with chronic stress exposure and plays a role in late pregnancy fetal maturation to the extra-uterine life. Therefore, as an additional analysis (Additional file 4: Table S3) we evaluated the glucocorticoid pathway exposure (indirectly measured by the GES) as the outcome variable. Prematurity, negative feelings during pregnancy and GAA were negatively associated with GES (Adj. R^2^ = 0.25).

## Discussion

Biological variability in fetal development and maturity in response to stress seems to occur in a sex-specific manner [17, 31]. We aimed to evaluate whether GAA could be predicted by epigenetic scores of low-grade inflammation (i-ePGS) and glucocorticoid exposure (GES) considering maternal stress exposure variables, newborn anthropometry, and the sex moderation in these associations. The results of this study showed (1) Infants who presented higher i-ePGS, whose mothers were younger and more stressed about the gestation, presented higher GAA, independent of the fetus sex; (2) even when accounting for other variables, sex moderation was observed as the higher was the GAA the higher was the GES, specifically in girls. Furthermore, in both models the maternal history of mental disorder was negatively associated with GAA.

Glucocorticoid levels have been suggested as a biomarker of chronic stress but evidence linking self-reported maternal prenatal stress to maternal cortisol levels during pregnancy is inconsistent. In the large ABCD cohort of pregnant women to investigate determinants of pregnancy cortisol, they found that 32% of all variance in cortisol levels was explained by gestational age, maternal age, hour of day, parity, pre-pregnancy BMI, c-reactive protein, fetal sex, smoking behavior, self-reported sleep sufficiency, and employment. They suggest that maternal cortisol during pregnancy is mainly affected by biological and lifestyle factors, and that psychosocial stress in pregnancy might program the fetus through other mechanisms than altering maternal cortisol levels. However, maternal history of depression and its association with inflammatory markers during gestation is more consistent. Here, we adopted indirect markers of prenatal glucocorticoid and inflammation exposure, obtained by DNAm levels in sensitive CpGs sites. Previously, using two different cohorts, prenatal anxiety was not associated with the epigenetic proxy of glucocorticoid exposure [6]. In our findings, maternal feeling related to pregnancy was not a GAA predictor in the model considering the GES, being significant only in the i-ePGS model, even though the presence of maternal history of mental disorder contributed to both models. Previous findings suggest that maternal-placental-fetal stress physiology partly mediates the observed outcomes via glucocorticoids, but inflammation, among other factors, is also relevant. Perhaps the relationship with glucocorticoids is in fact not directly linked to the mother's stress status, but regardless of the lack of association with some indicators of psychosocial stress, the overexposure to cortisol has both short and long-term effects on the conceptus.

GAA was positively associated with biomarkers of metabolic imbalances during pregnancy, such as increased homocysteine levels, reported in the sample from the Generation R study. Among the anthropometric outcomes, cephalic circumference was positively associated with GAA. No other neonatal measures were related to GAA. Head circumference was previously associated with GAA in a South African birth cohort study [11]. Cephalic circumference is a known clinical measurement to predict health outcomes, including neurodevelopment. Furthermore, a higher z-score at birth was positively associated with general cognitive scores and presented a protective association with ADHD-DSM-IV total symptoms, although not with cognitive scores.

There is a growing body of evidence showing that prenatal stress in humans leads to sexually dimorphism at birth and behavioral outcomes in the offspring [14]. Additionally, there is a hypothesis that GAA is a consequence of the fetal ability to react to an adverse environment and promote an adaptive response to rush the cell maturation/differentiation. Models that implicate variation in maturation, placental functioning, and the neuroendocrine milieu as potential contributors have been explored. The glucocorticoid pathway presents a role in cell maturation and differentiation, and we considered it as a possible pathway that could promote epigenetic maturity in a sex dependent manner in our analysis. Specifically prenatal exposure to maternal cortisol, exerts distinct influences on male and female development. Sex differences have been reported in the association between prenatal exposures to cortisol and maternal pregnancy-related anxiety and amygdala volume. Maternal cortisol concentration at 31 gestational weeks was most strongly associated with altered neural connectivity in girls, suggesting that girls exhibit an adaptive response by increasing the neural network connectivity necessary for maintaining homeostasis and efficient brain function across the lifespan.

Decelerated gestational age was reported in mothers with depression in fetus from both sexes but significantly predicted adverse outcomes in boys. Boys—but not girls—who exhibited lower GAA exhibited more internalizing problems, such as anxious-depressive symptoms or somatic complaints, at follow-up. The study replicated the association between maternal prenatal depression and child epigenetic GA deceleration and suggested that it may be driven by maternal serotonin reuptake inhibitors used during pregnancy. This might suggest that boys do not have the same pace for cell maturation, and it may represent a risk factor for neurodevelopment. It was also reinforced by our data that girls but not boys accelerate their biological age when interpreting an intrauterine adverse environment. Although it is beyond our analysis, it is possible that the indirect measurement of glucocorticoid exposure adopted here could reflect a faster fetal maturation process in girls. Knight et al. [28] who observed that infants with higher gestational age acceleration were less likely to receive surfactant, supporting the hypothesis that GAA is a marker of developmental maturity, including lung maturation. Furthermore, they found sex-specific differences in GAA, with higher means in girls. A previous study from the same group also showed that GAA in female infants and that antenatal betamethasone was associated with increased gestational age acceleration, which is consistent with antenatal corticosteroids being used to accelerate maturity before birth [12]. Although, an epigenetic methylation index of glucocorticoid exposure predicted age acceleration in males, but in different stages of life, which might represent different metabolic avenues taken given a stress exposure [6].Understanding the triggers in early life stages of vulnerability for age acceleration can provide valuable interpretation of the routes and deviations of metabolic pathways in a stressful environment. However, we are beginning to understand the biological interpretation and predictors of age acceleration in early life.

A number of limitations of the current study should be acknowledged. Our sample size can limit the statistical power of the association analyses presented. Given the descriptive nature of the study, we also cannot infer what is the cause and what is the effect. Additionally, we did not have a clinical measure of anxiety or depression for our sample, only a self-reported scale of perceived stress related to pregnancy, and those are important components in the dimension of maternal psychosocial stress. Furthermore, the mental disorder data was obtained by self-report confirmed by questioning the presence or absence of the use of psychiatric drugs, which also becomes a limitation in the context of the diagnosis of mental disorders. Moreover, exposure trajectories may provide valuable contributions to understanding the time-specific vulnerability to GAA. Finally, it would be interesting not only to analyze sex interactions but also stratify the analyses by sex.

## Conclusions

These findings add to the evidence that triggers of GAA present sex-specificity. The epigenetic score i-ePGS, an indirect measure of low-grade inflammation, was a predictor of GAA in our model for both sexes. Interestingly, the GES, an indirect measure of glucocorticoid exposure, seems to be more important to GAA in girls. Thus, this study supports the evidence of sex-specificity in stress response [14, 17, 28], suggesting that girls accelerate the maturation in face of adverse conditions and glucocorticoid exposure.

## Methods

### Participants

The participants came from the Western region of Sao Paulo city, where 6,200 women were enrolled at the university Hospital HU-FMUSP from 2012–2014 (Regiao Oeste Cohort, ROC). During the post-partum hospital stay, a questionnaire to approach social stress and toxic exposures during pregnancy (smoke and drug use) was administered to mothers enrolled in the cohort. During delivery, we collected blood from the umbilical cord of 96 women. All women gave written informed consent to participate in the study.

### Prenatal stress exposures

The postpartum questionnaire contained socioeconomic information as well as potentially stressful variables: self-reported perceived stress in relation to pregnancy (scale 1–5: ultimately, abortion was considered), alcohol/drug abuse, smoking (yes or no), pre-pregnancy body mass index (BMI, as kg/m^2^), gestational weight gain and the self-report of mental disorder (yes or no, considered if the patient reported use of psychiatric medication) and other antenatal complications.

### Obstetric and anthropometry data at birth

Medical records from the University Hospital were used to collect obstetric and anthropometric data at birth. As a hospital protocol, 24 h after birth, the gestational age is calculated by the Capurro method, as well as the new ballard score. The following anthropometric measurements were collected at birth: weight (g), length (cm), and abdominal, thoracic, and head circumferences. The sex-specific growth patterns curves from the World Health Organization (WHO) and the Anthro v3.2.2 software (available at: https://www.who.int/childgrowth/software/en/) were used to calculate the Z scores of the following indices: Length/Age (L/A), Weight/Age (W/A), Body Mass Index/Age (BMI/A), and Head circumference/Age.

### Cord blood sampling and methylation analysis

Samples were processed as previously described [30]. Briefly, DNA was extracted from umbilical cord blood samples using QIAamp DNA Blood Mini Kit (Qiagen, Hilden, Mettmann, Germany), according to the manufacturer’s instructions. DNA was bisulfite-converted using the EZ DNA Methylation kit (Zymo Research, Irvine, California, USA), according to manufacturer’s instructions and hybridized in the Illumina Infinium HumanMethylation450 BeadChip array (Illumina Inc., San Diego, California, USA). Raw data was extracted by iScan SQ scanner (Illumina) with GenomeStudio software (v.2011.1), using the methylation module v.1.9.0 (Illumina), into IDAT files used for further analyses. Raw data files were normalized using Quantile and corrected for cell composition using a cord blood dataset as implemented in minfi. Multidimensionality graphs (MDS) were also used to obtain the biological variability of the data. The X and Y chromosome probes were excluded to avoid sexual identity bias. Non-specific probes were excluded due to the high probability of co-hybridization. Single Value Decomposition method (SVD) identified technical bias (slide), which was corrected using ComBat. Probes from HM450K were annotated according to their nearest genes using FDb.InfiniumMethylation.hg19 package. Our main interest here with DNAm data was obtaining the epigenetic clock (EC).

### Epigenetic clock to estimate gestational age and gestational age acceleration (GAA)

The epigenetic age of each cord blood sample was estimated from raw beta values using two previously published epigenetic clock (EC) methods developed by Knight et al. [24] and by Bohlin et al. [23]. The ECs consist of an algorithm developed from the methylation data on CpGs sites of the Illumina 450 K array, which gives an estimate of the gestational age in samples of umbilical cord blood. The gestational epigenetic clocks can be compared with clinical/chronological GA to determine gestational age acceleration (GAA). Therefore, age acceleration was obtained from the residuals of the linear regression model between EC and clinical GA.

### Glucocorticoid (GES) and inflammatory (i-ePGS) epigenetic risk score

Previously, McGill et al. [6] using two cohorts from the Netherlands (Basal Influences on Baby Development) and Singapore (Growing Up in Singapore Towards Healthy Outcomes) created a glucocorticoid index exposure from 24 CpGs sites. The authors adopted an unweighted score defined as the sum of DNA methylation (standardized beta values) across previously found glucocorticoid sensitive CpGs. Here we adopted this Glucocorticoid Epigenetic Score (GES) representing the mean methylation levels of these 24 CpGs sites. We also adopted an Inflammation-related epigenetic polygenic risk score (i-ePGS) suggested by Barker et al. [7] as a biomarker of low-grade inflammation in a developmentally sensitive framework.

### Statistical analysis

The database processing, statistical analyzes and graphs construction were performed using the R software (version 4.1.3). The normality of the data was assessed by the Shapiro–Wilk test. For comparison of continuous and discrete variables, Student's t and Chi-square tests were applied. For nonparametric data, the Wilcoxon test was used. We first calculated the GAA residuals and bivariate analyses were used to explore unadjusted associations between GAA and all environmental exposures variables collected here. Linear multiple regression models were applied to investigate the i-ePGS, GES, variables that are associated with psychosocial stress and neonatal anthropometry as predictors of GAA. Considering that cell-type heterogeneity can confound the associations between DNAm and phenotypes we controlled all analyses by cell heterogeneity. Additionally, as our hypothesis relies on sex specificity stress response, we tested sex interactions. The multivariable regression model was obtained by retaining variables with at least a trend-level difference (*p* < 0.2) at the bivariate level. Furthermore, as GES and i-ePGS represent different stress-related biomarkers and partially independent pathways, we included each epigenetic score in separate models, using the same co-variables. We adopted the conventional *p* value < 0.05 cut-off for statistical significance.

## Supplementary Information

**Additional file 1**. **Figure S1**. Correlation between infant variables (anthropometric, i-ePGS, glucocorticoid sensitive mean CPGs and obstetric outcomes. HCZ= head circumference for age, HAZ = height for age, BAZ = BMI for age, DNAmGA = DNA methylation Age by Bohlin et al., GA = gestational age, GAA = gestational age acceleration, GES = glucocorticoid epigenetic score, i-ePGS = Inflammation-related epigenetic polygenic risk score. **Figure F2**. Correlation between mother variables, i-ePGS, glucocorticoid score and obstetric outcomes. GES = glucocorticoid epigenetic score, i-ePGS = Inflammation-related epigenetic polygenic risk score, GAA = gestational age acceleration.

**Additional file 2: Table S1**. Summary descriptive table by groups of GAA.

**Additional file 3: Table S2**. Bivariate analysis with GAA as outcome and newborn anthropometry as interest.

**Additional file 4: Table S3**. Multivariate model with GAA as outcome and GES as epigenetic risk exposure.

## Data Availability

The HumanMethylation450 BeadChip data set from this study is available in NCBI Gene Expression Omnibus (GEO) under accession number GSE85042. All data generated in this study are included in the Supplementary Information.

## Abbreviations

BMI: Body mass index
CI: Confidence interval
CpG: Cytosine-phosphate-guanine
DNAm: DNA methylation
EC: Epigenetic clock
GA: Gestational age
GAA: Gestational age acceleration
GES: Glucocorticoid exposure epigenetic score
i-ePGS: Low-grade inflammation epigenetic score
