# Supplementary material for: Gestational age acceleration is associated with epigenetic biomarkers of prenatal physiologic stress exposure
Source: Clin Epigenetics. 2022 Nov 28;14:152. doi: 10.1186/s13148-022-01374-9 (PMC9703828; doi:10.1186/s13148-022-01374-9)
Supplement: Supplementary file 1 — Additional file 1. Figure S1. Correlation between infant variables (anthropometric, i-ePGS, glucocorticoid sensitive mean CPGs and obstetric outcomes. HCZ= head circumference for age, HAZ = height for age, BAZ = BMI for age, DNAmGA = DNA methylation Age by Bohlin et al., GA = gestational age, GAA = gestational age acceleration, GES = glucocorticoid epigenetic score, i-ePGS = Inflammation-related epigenetic polygenic risk score. Figure F2. Correlation between mother variables, i-ePGS, glucocorticoid score and obstetric outcomes. GES = glucocorticoid epigenetic score, i-ePGS = Inflammation-related epigenetic polygenic risk score, GAA = gestational age acceleration. [file 13148_2022_1374_MOESM1_ESM.pdf]

Supplementary Figures

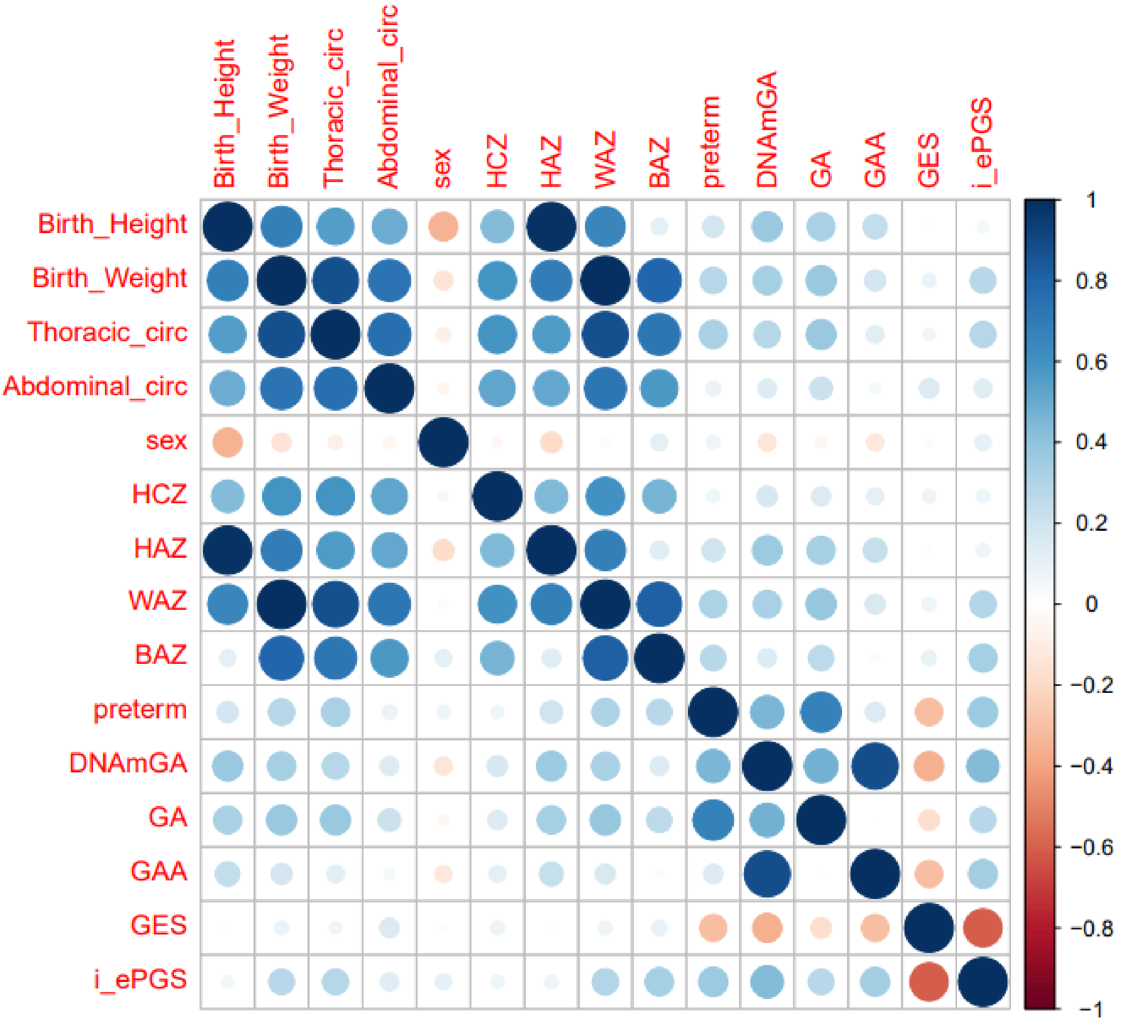

Supplementary Figure 1. Correlation between infant variables (anthropometric, i-ePGS, glucocorticoid sensitive mean CPGs and obstetric outcomes. HCZ= head circumference for age, HAZ = height for age, BAZ = BMI for age, DNAmGA = DNA methylation Age by Bohlin et al., GA = gestational age, GAA = gestational age acceleration, GES = glucocorticoid epigenetic score, i-ePGS = Inflammation-related epigenetic polygenic risk score.

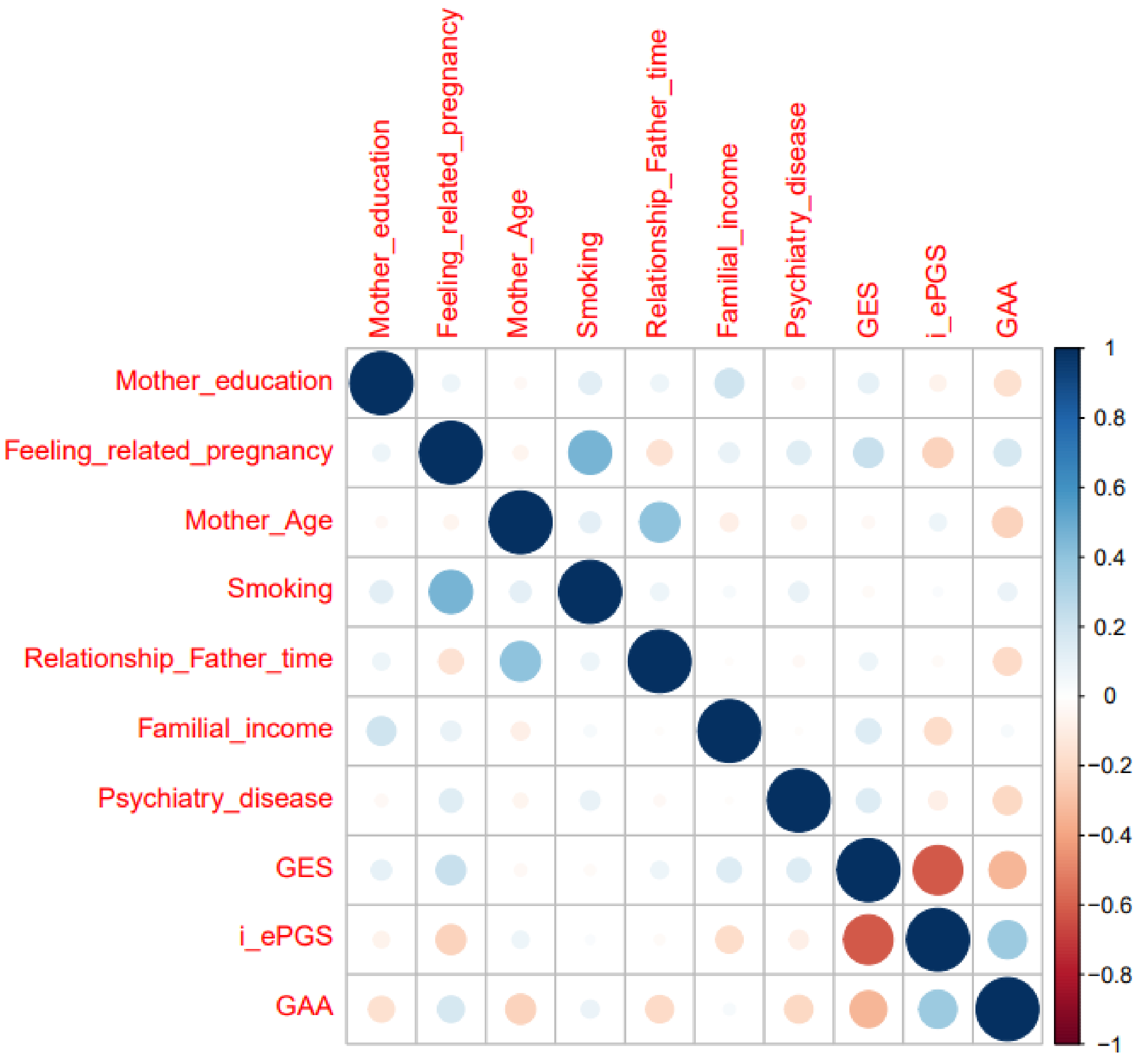

Supplementary Figure 2. Correlation between mother variables, i-ePGS, glucocorticoid score and obstetric outcomes. GES = glucocorticoid epigenetic score, i-ePGS = Inflammation-related epigenetic polygenic risk score, GAA = gestational age acceleration.
