# Supplementary material for: Gestational age acceleration is associated with epigenetic biomarkers of prenatal physiologic stress exposure
Source: Clin Epigenetics. 2022 Nov 28;14:152. doi: 10.1186/s13148-022-01374-9 (PMC9703828; doi:10.1186/s13148-022-01374-9)
Supplement: Supplementary file 2 — Additional file 2: Table S1. Summary descriptive table by groups of GAA. [file 13148_2022_1374_MOESM2_ESM.pdf]

Supplementary Table 1: Summary descriptive table by groups of GAA.

	**Decelerated** *N=37*	**Accelerated** *N=45*	**p-value**
**Prenatal exposures**			
Mother age	26.6 (6.45)	25.3 (7.19)	0.381
Pre-pregnancy BMI	24.9 (5.85)	25.3 (4.59)	0.771
Educational level (mother):			
1 -2	20 (55.56%)	25 (59.7%)	0.233
3 - 4	13 (37.1%)	16 (38.1%)	
5	3 (8.33%)	1 (2.38%)	
Mother feeling during pregnancy	1.95 (1.18)	1.73 (0.95)	0.357
Drugs and/or Alcohol abuse:			
No	24 (64.9%)	35 (77.8%)	0.295
Yes	13 (35.1%)	10 (22.2%)	
Psychiatry drugs during gestation:			
No	39 (95.1%)	37 (90.2%)	0.675
Yes	2 (4.88%)	4 (9.76%)	
Time in relationship with the father	57.4 (45.4)	69.0 (55.0)	0.309
Smoking:			
No	9 (22.0%)	7 (17.1%)	0.781
Yes	32 (78.0%)	34 (82.9%)	
Familial Income	4.52 (0.67)	4.22 (1.14)	0.182
**Newborn Outcomes**			
Gestational Age (capurro)	39.3 (1.20)	39.4 (1.88)	0.772
DNAmGA Bohlin et al. (weeks)	39.3 (0.66)	39.5 (0.68)	0.155
i-ePGS	-0.03 (0.01)	-0.03 (0.01)	0.046
GES	-1.02 (0.34)	-0.88 (0.36)	0.076
Sex:			
Boys	20 (48.8%)	17 (41.5%)	0.657
Girls	21 (51.2%)	24 (58.5%)	
Length	48.9 (1.92)	48.2 (2.28)	0.194
Weight	3410 (535)	3224 (447)	0.092
Thoracic circumference	33.6 (1.88)	32.9 (1.50)	0.074
Abdominal circumference	32.6 (2.45)	32.1 (1.86)	0.338

Cephalic circumference/Age	0.42 (1.04)	0.04 (0.88)	0.082
Weight/Height	0.82 (1.30)	0.60 (1.14)	0.430
Height/Age	-0.35 (0.98)	-0.65 (1.16)	0.214
Weight/Age	0.19 (1.08)	-0.16 (0.95)	0.120
BMI/Age	0.56 (1.27)	0.30 (1.03)	0.306
