# Supplementary material for: Gestational age acceleration is associated with epigenetic biomarkers of prenatal physiologic stress exposure
Source: Clin Epigenetics. 2022 Nov 28;14:152. doi: 10.1186/s13148-022-01374-9 (PMC9703828; doi:10.1186/s13148-022-01374-9)
Supplement: Supplementary file 3 — Additional file 3: Table S2. Bivariate analysis with GAA as outcome and newborn anthropometry as interest. [file 13148_2022_1374_MOESM3_ESM.pdf]

Supplementary Table 2: Bivariate analysis with GAA as outcome and newborn anthropometry as interest.

	**Gestational Age Acceleration (GAA)**		
	**GAA**		
Predictors	*Estimates*	*CI*	*p*
BAZ	0.02	-0.17 – 0.22	0.822
WAZ	0.16	-0.05 – 0.38	0.140
HAZ	-0.15	-0.88 – 0.59	0.689
Sex	-0.07	-0.55 – 0.40	0.760
HAZ * sex	0.22	-0.21 – 0.65	0.319
HCZ	0.12	-0.11 – 0.35	0.295
Thoracic circ	0.07	-0.05 – 0.20	0.253
Abdominal circ	0.02	-0.08 – 0.13	0.659
