# Supplementary material for: Gestational age acceleration is associated with epigenetic biomarkers of prenatal physiologic stress exposure
Source: Clin Epigenetics. 2022 Nov 28;14:152. doi: 10.1186/s13148-022-01374-9 (PMC9703828; doi:10.1186/s13148-022-01374-9)
Supplement: Supplementary file 4 — Additional file 4: Table S3. Multivariate model with GAA as outcome and GES as epigenetic risk exposure. [file 13148_2022_1374_MOESM4_ESM.docx]

| **Supplementary Table 3.** Multivariate model with GAA as outcome and GES as epigenetic risk exposure | | | |
| --- | --- | --- | --- |
|  | **GES** | | |
| *Predictors* | *Estimates* | *CI* | *p* |
| (Intercept) | -0.04 | -1.01 – 0.94 | 0.942 |
| GAA | -0.15 | -0.25 – -0.05 | **0.005** |
| HCZ | 0.03 | -0.08 – 0.14 | 0.597 |
| WAZ | 0.07 | -0.03 – 0.18 | 0.166 |
| Prematurity | -0.52 | -0.91 – -0.14 | **0.009** |
| Sex (Girls) | -0.00 | -0.18 – 0.17 | 0.959 |
| Smoking | -0.08 | -0.31 – 0.15 | 0.501 |
| Psychiatry disorder | 0.06 | -0.25 – 0.37 | 0.689 |
| Relationship Father time | 0.00 | -0.00 – 0.00 | 0.464 |
| Mother education | 0.03 | -0.06 – 0.12 | 0.555 |
| Familial income | 0.02 | -0.08 – 0.11 | 0.737 |
| Mother Age | -0.01 | -0.02 – 0.01 | 0.283 |
| Feeling related pregnancy | 0.11 | 0.03 – 0.20 | **0.011** |
| Observations | 68 | | |
| R^2^ / R^2^ adjusted | 0.374 / 0.238 | | |
|  |  |  |  |
